# Cognitive Changes and Quality of Life in Neurocysticercosis: A Longitudinal Study

**DOI:** 10.1371/journal.pntd.0001493

**Published:** 2012-01-31

**Authors:** Mitchell T. Wallin, E. Javier Pretell, Javier A. Bustos, Marianella Caballero, Mercedes Alfaro, Robert Kane, Jeffrey Wilken, Cynthia Sullivan, Timothy Fratto, Hector H. Garcia

**Affiliations:** 1 Neurology Service, Department of Veterans Affairs Medical Center, Washington, D.C., United States of America; 2 Department of Neurology, Georgetown University Medical School, Washington, D.C., United States of America; 3 Department of Neurology, Hospital Alberto Sabogal, Callao, Peru; 4 The Cysticercosis Working Group in Peru, Lima, Peru; 5 Department of Microbiology, School of Sciences, and Center for Global Health-Tumbes, Universidad Peruana Cayetano Heredia, Lima, Peru; 6 Cysticercosis Unit, Department of Transmissible Diseases, National Institute of Neurological Sciences, Lima, Peru; 7 Applied Neuropsychological Services, P.C., Centreville, Virginia, United States of America; 8 Department of Psychology, Walter Reed Army Medical Center, Washington, D.C., United States of America; 9 Washington Neuropsychology Research Group, Fairfax, Virginia, United States of America; University of Washington, United States of America

## Abstract

**Background:**

Few studies have focused on the cognitive morbidity of neurocysticercosis (NCC), one of the most common parasitic infections of the central nervous system. We longitudinally assessed the cognitive status and quality of life (QoL) of patients with incident symptomatic NCC cases and matched controls.

**Methodology/Principal Findings:**

The setting of the study was the Sabogal Hospital and Cysticercosis Unit, Department of Transmissible Diseases, National Institute of Neurological Sciences, Lima, Peru. The design was a longitudinal study of new onset NCC cases and controls. Participants included a total of 14 patients with recently diagnosed NCC along with 14 healthy neighborhood controls and 7 recently diagnosed epilepsy controls. A standardized neuropsychological battery was performed at baseline and at 6 months on NCC cases and controls. A brain MRI was performed in patients with NCC at baseline and 6 months. Neuropsychological results were compared between NCC cases and controls at both time points. At baseline, patients with NCC had lower scores on attention tasks (p<0.04) compared with epilepsy controls but no significant differences compared to healthy controls. Six months after receiving anti-parasitic treatment, the NCC group significantly improved on tasks involving psychomotor speed (p<0.02). QoL at baseline suggested impaired mental function and social function in both the NCC and epilepsy group compared with healthy controls. QoL gains in social function (p = 0.006) were noted at 6 months in patients with NCC.

**Conclusions/Significance:**

Newly diagnosed patients with NCC in this sample had mild cognitive deficits and more marked decreases in quality of life at baseline compared with controls. Improvements were found in both cognitive status and quality of life in patients with NCC after treatment.

## Author Summary

Neurocysticercosis (NCC) is one of the most common parasitic infections of the central nervous system. Cognitive changes have been frequently reported with this disease but have not been well studied. Our study team recruited a group of new onset NCC cases and a matched set of healthy neighborhood controls and new onset epilepsy controls in Lima, Peru for this study. A neuropsychological battery was administered at baseline and at 6 months to all groups. Brain MRI studies were also obtained on NCC cases at baseline and at 6 months. Newly diagnosed patients with NCC had mild cognitive deficits and more marked decreases in quality of life at baseline compared with controls. Improvements were found in both cognitive status and quality of life in patients with NCC after treatment. This study is the first to assess cognitive status and quality of life longitudinally in patients with NCC and provides new data on an important clinical morbidity outcome.

## Introduction

Neurocysticercosis (NCC) is caused by an infection of the human central nervous system (CNS) by the larval stage of the pork tapeworm *Taenia solium*. NCC is one of the most common parasitic infections of the human CNS and has become an increasingly important emerging infection in the United States [1]. It is the most common cause of symptomatic epilepsy worldwide [2], [3].

NCC is a dynamic, pleomorphic disease due to the variety of locations, numbers, and stages of lesions within the individual host [4]. The disease may mimic many neurologic syndromes, and a uniform presentation has not been described. Seizures are the most commonly reported symptom at presentation, occurring in up to 50–80% of patients [5], [6]. Headaches and focal neurologic deficits are also common.

Patients with NCC often display cognitive impairment. Mild to moderate cognitive dysfunction has been reported in up to 88% of NCC patients [7]. Dementia, defined through bedside testing, has been found in 6 to 15% of NCC patients in large clinical series [5], [8]. Levav, et al was the first group to use a standardized neuropsychological battery in assessing the cognitive status of patients with NCC [9]. They found significant deficits in motor control and impulsivity in NCC cases compared to controls. More recently, Ciampi De Andrade, et al used a comprehensive neuropsychological battery to evaluate a group of patients with NCC and matched healthy and epilepsy controls [10]. The group found impairment in multiple cognitive domains in patients with NCC prior to treatment as compared to both groups of controls. The use of antiepileptic drugs and seizures did not account for the cognitive abnormalities.

The studies that have utilized standardized cognitive batteries in NCC have been cross-sectional in nature. Because NCC is a heterogeneous disorder with great differences in disease duration, clinical features, and disability, it is important to control for as many of these factors as possible. Several unanswered questions remain about the temporal nature of cognitive dysfunction in the disease. For example, how do cognitive changes evolve over time? Are there risk factors that influence the course? Finally, what is the impact of NCC on the quality of life of patients with this infection?

We used a comprehensive standardized battery to assess the cognitive status of patients with newly diagnosed NCC at baseline and at six months. A group of demographically matched healthy neighborhood controls and epilepsy controls were recruited for comparison. Similar to NCC cases, the epilepsy controls had new onset seizures to adjust for the effects of antiepileptics on cognitive performance.

## Methods

### Ethical Considerations

This study was approved by the Institutional Review Board (IRB) at the of the Universidad Peruana Cayetano Heredia, the Instituto Nacional de Ciencias Neurologicas, and the Hospital Alberto Sabogal all in Lima, Peru, as well as the IRBs of Georgetown University and the VA Medical Center, both in Washington, DC. All subjects enrolled in the study provided written informed consent.

### Study Participants

A total of 14 patients with NCC were recruited at the Sabogal Hospital and Cysticercosis Unit, Department of Transmissible Diseases, National Institute of Neurological Sciences in Lima, Peru. To make the baseline time point consistent across cases, patients with NCC were recruited within six months of a new-onset seizure associated with parenchymal NCC. These incident symptomatic cases had a positive serologic enzyme-linked immunotransfer blot (EITB) assay and met standard diagnostic criteria for NCC [11]. Seizures must have been controlled with standard antiepileptic drugs (AED) for inclusion. Exclusion criteria for cases included central nervous system disease not related to NCC, active alcohol or drug abuse, primary psychiatric disease prior to developing NCC, disorders of vision that would preclude cognitive testing and pregnancy.

For comparison, 14 healthy neighborhood controls were recruited along with the NCC cases. Healthy controls were recruited from the neighborhood of the patients with NCC and were matched to cases on age (+/−10 years) and education (+/−5 years). Controls were EITB negative, had no chronic health conditions and had similar exclusion criteria as cases.

Epilepsy controls were also recruited at the Sabogal Hospital after new onset seizures (N = 7). Epilepsy was defined according to revised criteria from the International League against Epilepsy [12]. All seven epilepsy controls had a brain CT prior to enrollment which was normal. Seizures were both generalized and focal and their epilepsy was classified as unknown etiology. Seizures were controlled in all subjects with standard AEDs. All epilepsy controls were EITB negative, and met exclusion criteria as per NCC cases.

### Procedures

All patients participating in the study had a screening history and physical at baseline. Patients with NCC were treated with a standard 10-day course of albendazole [13] (15 mg/kg/day divided bid) and started on an AED regime that was consistent throughout the 6 month follow-up period. Similarly, patients with new onset seizures were started on a standard AED regime during the study period. Neuropsychological testing was performed at a mean interval of 8 weeks from starting AED medications for new onset epilepsy cases and a mean interval of 11 weeks after completing antihelminthic medication and initiating AEDs for new onset NCC cases.

A brain MRI was performed at baseline and month 6 for all NCC patients at a private radiological center on a 1.5-Tesla unit, as in previous trials [13], [14]. The protocol included T-1 weighted axial with and without intravenous gadolinium (0.1 mmol/kg), and T2-weighted and FLAIR axial studies. A radiologist experienced with NCC cases read each imaging study to determine the number, stage and location of CNS parenchymal cysticerci. Cure rate was determined by the number of patients who had resolution of cysts by brain MRI at 6 month follow-up.

### Neuropsychological Testing Battery

The neuropsychology testing battery for the current study, administered at baseline and 6 month follow-up to all patients, was chosen to tap into the various domains of cognitive functioning thought to be affected by NCC. It is sensitive to lapses in attention, decreased visual and motor processing, and problems with working memory. It was developed in consultation with Dr. Mirsky based on his experience of testing NCC patients in Ecuador [9]. In addition, a standard quality of life (QoL) instrument, the SF-36, was included in the battery. The tests in the battery are listed and referenced under their cognitive domain in **Table 1**. All tests were administered by a single native Spanish speaking psychology technician using standard Spanish translations of each test. The instructions for tests were given to patients in Spanish using a standard script.

**Table 1:** Neuropsychological testing battery.

Neuropsychological Domain	Test
Vigilance/Sustained Attention	Kay Continuous Performance Test [27]
Processing Speed	Stroop Color & Word Test [28]
	Talland Letter Cancellation Test [29]
	#Trail Making Test (Parts A and B) [30]
	#WAIS-III [31]: Digit Symbol-Coding Test
Simple/Complex Attention	*WAIS-III [31]: Digit Span Test
	*WAIS-III [31]: Arithmetic Test
	WMS-III [32]: Mental Control
Learning/Encoding	¶WMS-III [32]: Logical Memory I
	¶RAVLT [33] Trials 1–5
Memory/Recall	§WMS-III [32] Logical Memory II
	§RAVLT [33] Delayed Recall
Problem Solving/Reasoning	Wisconsin Card Sorting Test [34]
	Ravens Standard Progressive Matrices [35]
General Cognitive Function	Mini-Mental Status Examination [36]
Affect	Beck Depression Inventory [37]
	Beck Anxiety Inventory [38]
Quality of Life	SF-36 [39]

*Attention composite tests;

# Processing speed composite tests;

**¶:** Learning composite tests;

**§:** Memory composite tests (see Table 3).

Abbreviations: WAIS: Wechsler Adult Intelligence Scale, WMS: Wechsler Memory Scale, RAVT: Raven's Progressive Matrices and Vocabulary Scales, SF-36: Short Form-36.

### Statistical Analysis

Group means for individual neuropsychological tests at each visit (baseline, and 6 months) were calculated for NCC cases and controls. SF-36 transformed scale scores were calculated from the raw scale scores. A p-value≤0.05 was considered significant. Differences between groups were assessed by the χ2 test for categorical variables and the t-test for continuous variables. Because there are no national normative data for Peru, results for the NCC cases were assessed in the context of healthy controls, and epilepsy controls.

Due to the number of individual neuropsychological tests administered, a composite score was computed for the assessed cognitive domains. The tests comprising the composite scores for the major cognitive domains of attention, processing speed, learning and memory are defined in **Table 1**. Additional tests covering the following neuropsychological areas are also included in the battery: a) vigilance/sustained attention; b) general cognitive function; c) affect; and d) quality of life. These tests were not part of the composite scores.

The composite score is made up of the individual tests within the specified domain and is highly correlated to the individual test scores. Using composite scores focuses the analysis on specific cognitive domains and reduces the likelihood of making Type 1 errors by minimizing the number of variables in the analysis. Composite scores also attenuate ceiling and floor effects of individual test scores and are helpful in finding longitudinal changes when there is heterogeneity in cognitive function [15], [16]. In each case, raw scores were converted into z-scores based on US population-based normative data for each standardized test. The z-scores were averaged within each domain yielding a standardized measure of a participant's (both cases and controls) average performance on key measures within each cognitive domain. For example, the memory composite score was calculated by adding the individual z-scores of the Wechsler Memory Scale -III Logical Memory II and the Rey Auditory Verbal Learning Test delayed recall and dividing by 2, the total number of scores in the index.

We used both one-way and multiple ANOVA to examine how NCC cases performed on various measures of neuropsychological function and QoL as compared with controls at each testing time point. We also compared changes in neuropsychological testing scores longitudinally within the same group using t-tests. For multiple regression, demographic and variables were entered as covariates in the standard linear models. Adjustments were not made for multiple comparisons. Within the four composite cognitive domains, we considered a z-score below 1.5 standard deviations to be impaired. We compared the proportion of subjects that were impaired between groups at the baseline assessment. SPSS version 14 (Chicago, IL) was used for the statistical analysis.

## Results

There were no statistically significant differences in the major demographic variables (**Table 2**) among the NCC cases, neighborhood controls and epilepsy cases in this study. There were no clinical seizures during the study follow-up period in either the NCC cases or epilepsy controls. Patients with NCC and epilepsy remained on the initial antiepileptic therapy they were prescribed during the 6-month study follow-up period. There were no cases of hydrocephalus or increased intracranial pressure in the NCC group per neuroimaging at any time point in the study.

**Table 2:** Demographic and clinical summary of neurocysticercosis cases and controls.

Demographic & Clinical Variables	NCC Cases(N = 14)	Neighborhood Controls(N = 14)	Epilepsy Controls(N = 7)
Age (yrs at baseline), mean (SD)	28 (10)	28 (10)	30 (6)
Male sex (no.)	10	6	4
Education: n (%)			
0–8 yrs	5 (36)	1 (7)	1 (0)
9–12 yrs	3 (21)	6 (43)	3 (43)
>12 yrs	6 (43)	7 (50)	4 (57)
Occupational status:			
Unemployed: n (%)	0 (0)	0 (0)	0 (0)
Student: n (%)	5 (36)	2 (14)	0 (0)
Employed nonprofessional: n (%)	8 (57)	9 (64)	3 (43)
Employed professional: n (%)	1 (7)	3 (21)	4 (57)
Number of cysts (baseline)			
Total: median (IR)	2 (5.8)	-	-
Cysts w/o degeneration: median (IR)	1.5 (4.8)	-	-
Cysts with degeneration: median (IR)	1.0 (1.0)	-	-
Calcified cysts: median (IR)	0 (0)	-	-
Antiepileptic medication: n (%)			
Phenytoin	9 (64)	-	3 (43)
Carbamazepine	4 (29)	-	3 (43)
Clonazepam	0 (0)	-	1 (14)
Valproate & Carbamazepine	1 (7)	-	0 (0)
Baseline number of seizures: mean (SD)	2.7 (1.9)	-	2.1 (0.4)
Seizure type:			
Generalized: n (%)	6 (43)	-	6 (86)
Partial: n (%)	2 (14)	-	1 (14)
Both Generalized & Partial: n (%)	6 (43)	-	0 (0)

Abbreviations: SD: standard deviation, IR: interquartile range.

Comparisons were made among the groups (NCC, neighborhood healthy controls, and epilepsy controls) at baseline using multiple ANOVA (**Table 3**) with scores adjusted for age and education. At baseline, patients with NCC had lower Attention Composite Scores than the epilepsy controls (p<0.04). For other composite cognitive domains, patients with NCC generally scored lower than controls. However, there were no significant differences in baseline composite scores for learning, memory, processing speed or other neuropsychological tests when comparing NCC cases with epilepsy controls or NCC cases with neighborhood controls (all p≥0.13).

**Table 3:** Composite age and education-adjusted test scores for major cognitive domains at baseline and 6-month follow-up.

	Attention		Processing Speed		Learning		Memory	
Group	Baseline	Follow-up	Baseline	Follow-up	Baseline	Follow-up	Baseline	Follow-up
NCC	34.0 (9.0)**	35.5 (9.8)	31.5 (10.2)	33.0 (11.3)	37.7 (12.2)	41.3 (12.0)	39.7 (9.4)	40.5 (11.3)
Epilepsy Controls	44.0 (8.4)	43.8(6.5)	38.1 (5.2)	41.1 (6.3)	44.3 (7.0)	49.8 (6.4)	43.2 (6.3)	51.0 (5.7)
Neighborhood Controls	36.7 (7.2)	37.4 (3.9)	34.5 (11.4)	36.0 (9.0)	41.1 (8.9)	41.3 (7.4)	40.7 (7.6)	44.5 (5.8)

**Significant difference between NCC and Epilepsy groups cross-sectionally (p≤0.05). Standard deviation follows test scores in brackets.

The proportion of cognitively impaired patients with NCC at baseline within the composite domains of attention, processing speed, learning and memory was 57%, 57%, 50% and 36% respectively. Similarly, neighborhood controls had 43%, 43%, 29% and 29% of subjects impaired in the same respective cognitive domains. Epilepsy controls had 14% impairment in each of the composite domains. Overall, there was a greater trend for patients with NCC to be impaired in all four cognitive composites when compared with neighborhood controls and epilepsy controls.

QoL summary scores at baseline revealed a significant decrement in social function (p = 0.002) and borderline decreases in physical function (p = 0.067) and mental function (p = 0.072) in the NCC group compared with neighborhood controls (**Table 4**). Epilepsy controls had a significant QoL decrease in mental health function (p = 0.028) and borderline low social function (p = 0.065) compared with neighborhood controls. The number of seizures at onset were not significantly correlated with any baseline QoL summary score.

**Table 4:** SF-36 transformed scale scores for major quality-of-life outcomes at baseline and 6-month follow-up.

	SF-36 Physical Function		SF-36 Mental Health Function		SF-36 Social Function	
Group	Baseline	Follow-up	Baseline	Follow-up	Baseline	Follow-up
NCC	85.7 (4.4)*	91.3 (5.6)	57.4 (4.8)*	64.7 (5.1)	53.8 (5.6)** #	77.3 (5.3)#
Epilepsy Controls	85.0 (7.7)	90.0 (5.2)	50.3 (9.1)**	59.2 (5.4)	60.9 (10.3)*	68.2 (6.4)
Neighborhood Controls	98.2 (0.9)	97.8 (1.7)	73.8 (4.2)	76.4 (5.1)	84.4 (4.7)	83.4 (6.2)

*Difference vs. Neighborhood Controls cross-sectionally (p≤0.07);

**Significant Difference vs. Neighborhood Controls cross-sectionally (p≤0.05);

# Significant difference within groups longitudinally (p≤0.05). Standard error follows transformed test scores in brackets.

QoL scores at the six month follow-up visit showed an overall trend for improvement across physical health, mental health and social function domains in both NCC and epilepsy groups (Table 4). There were no significant or borderline differences cross-sectionally between any group with all p values>0.1.

All groups were examined for cognitive changes over time (i.e., between baseline and 6-month follow-up testing). Within the cognitive battery of tests, NCC cases had significant improvement in psychomotor speed and working memory as assessed by the WAIS-III Digit Symbol Coding Subtest (p = 0.012) and by the Trail Making Test Part B (p = 0.016) between the baseline and 6-month follow-up sessions but there were no significant changes in any composite score (**Table 5**).

**Table 5:** Individual cognitive test scores within the processing speed composite at baseline and 6-month follow-up.

	Trail Making Test-Part B		WAIS-III Digit Symbol-Coding Test	
Group	Baseline	Follow-up	Baseline	Follow-up
NCC	31.3 (3.0)#	118.4 (19.8)#	50.6 (4.8)#	54.5 (5.2)#
Epilepsy Controls	40.0 (2.1)	73.0 (7.0)	66.9 (4.7)	71.7 (5.1)
Neighborhood Controls	36.4 (3.5)	93.6 (9.5)	59.0 (6.1)	62.7 (4.7)

# Significant difference within groups longitudinally (p≤0.05). Standard error follows mean test scores in brackets.

Impressive improvement in QoL for NCC cases was noted in the SF-36 domain assessing social function (p = 0.0062) over the 6-month follow-up period (Table 4). Despite improved scores for NCC and epilepsy groups between baseline and follow-up visits, there were no other significant or borderline longitudinal changes in the remaining QoL domains for any of the 3 patient groups (p values>0.4).

Among the demographic and clinical variables collected in the study, there were no significant longitudinal predictors of cognitive dysfunction or QoL in any group. Variables utilized in bivariate and multivariate statistical models included age, sex, education, occupation, number of CNS cysts, type of cysts, and cure rate of cysts.

## Discussion

This study demonstrated that patients with parenchymal NCC had relatively mild cognitive dysfunction shortly after presentation of seizures compared with two sets of control groups. Moreover, quality of life was significantly impaired in the NCC and epilepsy groups compared with healthy controls. Many reports of altered cognition in NCC have been related to hydrocephalus and intracranial hypertension which is not the case in this series.

After six months of follow-up, cognitive tasks involving attention/psychomotor speed and working memory improved in patients with NCC as did QoL. There were no significant demographic or clinical predictors of cognitive dysfunction or QoL in NCC cases or controls. This is the first study to longitudinally assess cognitive function and QoL in patients with NCC.

There are few publications that have specifically addressed cognitive changes in NCC. Most of the information on this topic comes from case series and anecdotal reports of NCC patients who experience psychiatric disturbances or mental status changes. How the cysticercotic CNS lesions exert their effects on cognition is unclear, but at least some of the mental status changes described in clinical reports could be explained by partial seizures, mass effect of cysts and increased intracranial pressure. The inflammatory immune response is critical in NCC pathogenesis but is poorly understood. CNS inflammation has been correlated with the cognitive dysfunction in a number of neurological conditions including multiple sclerosis and Alzheimer's dementia [17], [18]. Finally, the effects of AEDs on cognition in patients with epilepsy has been well studied [19]. Controlling for these effects with an epilepsy control group is prudent.

Longitudinal improvement trends in QoL were seen in patients with both NCC and epilepsy in this study. The mechanisms for decrements in QoL may be different than those producing cognitive dysfunction. Reduction in seizures which was seen in both the epilepsy and NCC groups over the follow-up period may be playing a role in improved QoL outcomes. We did not see a significant correlation between the number of seizure and QoL at baseline, however.

The earliest reports of mental status changes with NCC come from case series in the early 20^th^ century, where both clinical and pathologic aspects are described [1], [2]. Other early reports emphasize the importance of altered mental status as a presenting symptom of NCC [5]. McArthur made some relevant insights into British soldiers with neurocysticercosis in the 1930s:

“As well as gross disturbance which suggests diagnosis such as these above, there may be mental dullness, impairment of memory, temporary periods of disorientation, or a change in disposition so that a previously efficient soldier may become careless and untrustworthy. Indeed, Colonel Benson, commanding the military hospital, Millbank, has told me with some feeling that if any breach of ward discipline is reported, usually a cysticercosis patient proves to be the delinquent [20].”

Roselli et al. described a severe case of dementia associated with parenchymal NCC in a 15-year-old girl [21]. Treatment with praziquantal decreased intellectual deterioration but may have precipitated hydrocephalus. Latovski et al. reported five patients with NCC who presented to a New York hospital with signs of dementia, seizures and increased intracranial pressure [22]. All improved with surgical and/or medical therapy. Larger case series have reported the prevalence of dementia between 6% [3] and 16% [4]. Psychotic behavior has been the presenting symptom in individual NCC cases [5] and in 2% of 112 NCC cases from Houston, TX [23].

Forlenza et al. reported on the psychiatric and cognitive manifestations of NCC from a case series from Brazil [7]. All 38 NCC cases were examined cross-sectionally. The authors used the mini-mental state exam, the present state exam, and Strub and Black's mental status examination to assess cognitive function. Psychiatric disease was found in 66% of cases and cognitive decline in 88%. Mild to moderate cognitive deficits were more common than frank dementia. Interestingly, the number, type of brain lesions, use of corticosteroids, or epilepsy did not significantly correlate with the severity of psychiatric symptoms. The high rate of cognitive impairment was striking considering the relative insensitivity of the tests used.

The first study to use standardized cognitive testing in NCC patients and controls was published in 1995 by Levav et al [8]. The authors recruited a representative community sample of patients from a larger epidemiologic study on epilepsy and NCC in Ecuador. A total of 123 subjects agreed to participate in the study. A series of 11 standardized NP tests were administered to 20 patients with NCC and controls to measure five dimensions of neurocognitive function. The testing revealed that patients with NCC had significant deficits in attention and motor control compared with controls.

The most recent report on cognitive changes and NCC came from Ciampi De Andrade et al [10]. This Brazilian group recruited 40 treatment-naïve NCC cases, 49 healthy controls and 28 patients with cryptogenic epilepsy. The assessment of neuropsychological function was cross-sectional with NCC cases exhibiting significant impairment in executive function, memory, constructive praxis and verbal fluency compared with healthy controls and epilepsy controls. Similar to our study, there were no significant predictors of cognitive dysfunction based on clinical variables. In contrast to Ciampi De Andrade, et al, we did not find the degree of cognitive dysfunction in our NCC cases. However, the healthy controls in the study by Ciampi De Andrade, et al were made up in part of staff at the hospital and the epilepsy controls were established cases (vs. new cases) at the authors' institution. The controls used could have produced some bias in the cognitive testing results. For example, patients with established epilepsy may have accommodated to any untoward effects of anti-epileptic medications through modification of the dose or agent as compared to new onset epilepsy cases. We recruited healthy controls exclusively from the same neighborhood as cases and all epilepsy controls had new onset seizures of unknown etiology. These control groups mirrored the experience of NCC cases in our population.

To our knowledge, there have been no longitudinal QoL studies in patients with NCC. A recent cross-sectional study of QoL in patients with NCC from Mexico reported deficits in both mental and physical domains compared with controls [24]. Other neurological conditions that have demonstrated impairments in quality of life in Latin America include head trauma [25] and spinal cord injury [26].

There are limitations to our study. Our sample size was relatively small. This was due in part to the type of patients we recruited, new onset patients with NCC with seizures, and the longitudinal nature of the project. Second, we had relatively mild disease burden of parenchymal NCC with low number of cysts and few neurology symptoms outside of seizures. This is fairly typical of patients with NCC in endemic regions and the US. A more broad morbidity spectrum of NCC cases would likely provide a wider range of cognitive dysfunction. Finally, it would have been useful to have Peruvian neuropsychological test norms for comparison in this study. Unfortunately, these data are lacking for Peru and many Latin American countries. US norms were available for the majority of the cognitive tests and served as a reference for composite scores.

In summary, our study showed patients with parenchymal NCC have mild cognitive dysfunction along with more significant deficits in QoL compared with healthy neighborhood controls and epilepsy controls. Altered mental status and psychiatric presentations in NCC are the tip of the iceberg while more subtle cognitive dysfunction is quite common. Importantly, in our series, the cognitive and QoL deficits improve with time. Future epidemiologic and clinical studies of NCC should include cognitive function and QoL as outcome variables.
